# Favorite Prescriptions

**Published:** 1890-05

**Authors:** 


FAVORITE PRESCRIPTIONS.—*For Infantile Convulsions:* Dr. A. Jacobi, of New York, an authority upon diseases of children, first orders a purgative dose of calomel in cases of infantile convulsions, and then follows it in a few hours by:

| | | |
|---|---|---|
| ℞. | Chloral hydrat - - - - - | gr. iv. |
| | Potas. bromid. - - - - - | gr. viij. |
| | Aquæ | |
| | Syrupi - - - - - - | aa f℥j. |

M.—Sig. One dose for a child two years old.

